# The position along rhizome as a surrogate for root age: effect on fine root traits

**DOI:** 10.1093/aob/mcag103

**Published:** 2026-04-18

**Authors:** Adam Hrouda, Timothy Harris, Andrea Kučerová, Jitka Klimešová

**Affiliations:** Department of Botany, Faculty of Science, Charles University, Benátská 2, Praha 2 128 01, Czech Republic; Department of Experimental and Functional Morphology, Institute of Botany of the Czech Academy of Sciences, Dukelská 135, Třeboň 379 01, Czech Republic; Royal Botanical Gardens, Kew, Richmond, Surrey TW9 3AB, UK; Department of Experimental and Functional Morphology, Institute of Botany of the Czech Academy of Sciences, Dukelská 135, Třeboň 379 01, Czech Republic; Department of Botany, Faculty of Science, Charles University, Benátská 2, Praha 2 128 01, Czech Republic; Department of Experimental and Functional Morphology, Institute of Botany of the Czech Academy of Sciences, Dukelská 135, Třeboň 379 01, Czech Republic

**Keywords:** Fine root trait, herb, eudicot, intraspecific variation, rhizome, root ontogeny

## Abstract

**Background and Aims:**

Fine root morphology underlies the resource acquisition strategy of species, yet its development during root lifespan remains underexplored. Focusing on rhizomatous herbs, a promising system for root ontogeny research, we used root position along the rhizome as a proxy of root age and asked: how much do root traits differ between young and old fine roots?

**Methods:**

We collected whole clonal fragments of 19 temperate eudicot herbaceous species in Czechia, central Europe, always at two localities (per species) differing in moisture. We measured fine root morphological traits (in old and young parts of rhizomes), rhizome traits and above-ground biomass.

**Key Results:**

Root tissue density increased and specific root length decreased with fine root age in the studied species. Fine root diameter showed no overall change in response to root age but increased with root age in plants with longer rhizomes, possibly owing to greater age variation along longer rhizomes. The changes in fine root traits were small in magnitude in comparison to between-species differences in trait values and were not affected by site moisture or nutrient status.

**Conclusions:**

Our results suggest that fine root traits change during root lifespan, shifting towards higher root tissue density, larger diameter and smaller specific root length. This can lead to local trait variation within a single root system, possibly affecting small-scale processes, such as below-ground competition. On the level of species, this variation is relatively small and thus need not be reflected in root sampling methods.

## INTRODUCTION

Plants use fine roots mainly to obtain water and mineral nutrients from soil (Hodge *et al.*, 2009). These functions are achieved by a variety of strategies in terms of root morphology and structure. To compare between species or individuals, measurements of several fine root traits are often used, most commonly specific root length (SRL), root tissue density (RTD), root diameter and nitrogen (N) content (McGill *et al.*, 2006; Freschet *et al.*, 2021*b*). Plant strategies (i.e. trait syndromes) are usually defined at the level of species, building on the assumption of small or no intraspecific variation (McGill *et al.*, 2006; Violle *et al.*, 2012). However, some traits can vary substantially within species, sometimes reaching comparable magnitude to interspecific variation (Siefert *et al.*, 2015). Examining intraspecific variation in particular contexts can help us to understand the processes that might otherwise be left hidden (Violle *et al.*, 2012), such as differences between young and old plants or differences between organs at varying ontogenetic stages. This might prove even more relevant in fine roots, with the associated methodological difficulty of sampling. The fact that in root traits, researchers often work with part of a root system without knowing the context (i.e. its position in the root system) could lead to ignoring a systematic source of variation (Freschet *et al.*, 2021*a*). Understanding whether and how fine root traits within a root system vary is an essential step in strengthening our knowledge on plant form and function.

In perennial herbs, fine roots are often not annual (i.e. forming only one cohort per year), but typically have overlapping generations (Ryser and Kamminga, 2009; McCormack and Guo, 2014; Courchesne *et al.*, 2020). There is some evidence that fine root traits might change during root lifespan (Garbowski *et al.*, 2021; Havrilla *et al.*, 2021), yet current findings apply mostly to young seedlings only. To date, we have little information both on how root traits shift during fine root lifespan and on how this might translate into within-individual trait variation (between fine root cohorts). Exploring this variation can put current methods for root trait measurement to test and help us understand trait patterns at small spatial scales.

Ontogeny has been shown to affect multiple plant traits (Falster *et al.*, 2018; Henn and Damschen, 2021; Westerband *et al.*, 2021; Martínková et al., 2026), with most rapid trait changes usually happening at the earliest (seedling) stages of life (Henn and Damschen, 2021) and becoming slower with increasing plant age (Havrilla *et al.*, 2021). Changes in size and tissue density of plant organs over the lifespan of those organs have been shown for leaves (Mediavilla and Escudero, 2003), rhizomes (Lubbe *et al.*, 2023; da Silva *et al.*, 2026) and roots (Sun *et al.*, 2016). Studying changes in allocation to organs over their lifespan provides important information for understanding plant carbon economics (see Castorena *et al.*, 2022). Currently, incorporating fine root ontogeny into functional ecology studies presents several challenges. In most cases, destructive methods are used for root trait measurement, producing limited or no information on root age. There are several methods for the assessment of root traits with respect to root age, including rhizoboxes (Song et al., 2025), (mini)rhizotrons (Wells and Eissenstat, 2001; Li *et al.*, 2020; Hou *et al.*, 2024) or X-ray imaging (Mooney *et al.*, 2012). Still, there are various costs associated with the use of these methods; they can be expensive, laborious and require special equipment or a long duration. With these costs in mind, we decided to use a destructive method in this study. Instead of repeated root sampling (used by Sun *et al.*, 2016), we sampled the roots at a single time and used the position of roots along the rhizome to estimate their ontogenetic stage. This method takes advantage of the particular pattern of growth present in rhizomatous herbs.

Rhizomes are below-ground stems that produce new increments yearly, which are equipped soon after formation with fine adventitious roots (McCormack and Guo, 2014), persist for a species-specific time, then die together with their fine roots (Bartušková *et al.*, 2022). Rhizomes of herbs can live for several years (even a decade or more) (Klimeš *et al.*, 1997) whereas fine root lifespan is usually estimated to be from a few months to 2 years (Eissenstat and Yanai, 1997; McCormack and Guo, 2014; Courchesne *et al.*, 2020). Owing to a continuous cycling of roots (or root units; Sun *et al.*, 2016), the roots originating from a given part of a rhizome are not necessarily of the same age as the rhizome part itself. Also, we are seldom capable of determining the exact age of different parts of a rhizome (but see Bartušková *et al.*, 2022). That being said, we can positively distinguish between the young and old part of a rhizome (for whole-clone rhizome system analysis, see Tybjerg and Vestergaard, 1992,). Furthermore, the directional nature of rhizome growth (Yoshida *et al.*, 2016) leads to the age of roots increasing, on average, towards the old part. The result of these patterns is a root system where a temporal variable (i.e. root age) is also a function of space (i.e. position along the rhizome). Thus, rhizomatous herbs represent a promising study system for evaluating trait shifts during fine root lifespan.

In this study, we evaluated trait shifts during fine root lifespan using root position along the rhizome as a proxy of root age. Building on previous research on the development of root traits, we hypothesized that young roots will feature higher SRL and lower RTD and diameter in comparison to the old roots. To test the hypothesis, we analysed data on root morphology of 19 field-grown rhizomatous eudicot herbs of Central Europe. We also collected data about whole-plant parameters and site conditions to assess their effect on potential trait development during root lifespan.

## MATERIALS AND METHODS

### Study species and sites

Our study was conducted on 19 species of rhizomatous eudicot herbs (for species information, see Table 1). We chose the species based on lateral spread (species with lateral spread >20 cm year^−1^ were excluded owing to anticipated problems with excavation), their occurrence in the Czech Republic and their local abundance. Each of the species was sampled at two sites that were expected to differ in their soil moisture conditions. The total number of sites was 37. All sites were located in the Czech Republic. Sites were chosen based on expert knowledge of previous occurrence of target species and were used only if there was a sufficient number of plants of a given species (minimum population size was 30 individuals). On each site, a phytocenological relevé was done and based on the species presence or abundance, site conditions were estimated by Ellenberg indicator values (EIVs; Ellenberg, 1992; Chytrý *et al.*, 2018; for a table of EIVs of the collection sites, see Supplementary Data Table S1).

**Table 1.** List of species and selected trait values. Averages of trait values for the whole species are shown.

Species name	Abbreviation	Root traits			Rhizome traits		Above-ground traits	
		SRL (m g^−1^)	RTD (g cm^−3^)	Diameter (mm)	Rhizome DMC (%)	Rhizome length (mm)	Leaf biomass (g)	Stem + flower biomass (g)
*Agrimonia eupatoria* L.	agrimonia	28.5	0.31	0.40	37.0	62.4	5.79	6.08
*Alchemilla glaucescens* Wallr.	alchemilla	49.0	0.29	0.32	NA	NA	NA	NA
*Anemone nemorosa* L.	anemone	74.8	0.17	0.32	24.3	119.8	0.03	0.03
*Betonica officinalis* Delarbre	betonica	54.3	0.22	0.35	29.8	95.1	4.17	3.43
*Bistorta officinalis* Delarbre	bistorta	31.1	0.23	0.46	28.7	190.9	0.38	1.32
*Geum rivale* L.	geum	36.3	0.47	0.31	35.8	134.3	1.27	1.46
*Hepatica nobilis* Schreb.	hepatica	44.5	0.28	0.35	21.9	442.9	6.09	2.32
*Hieracium murorum* L.	hieracium	37.8	0.21	0.42	30.2	102.8	0.56	0.13
*Chelidonium majus* L.	chelidonium	32.2	0.27	0.43	29.5	95.0	0.26	0.42
*Leucanthemum ircutianum* DC.	leucanthemum	121.1	0.14	0.32	14.1	69.8	3.12	4.29
*Potentilla alba* Moench	pot_alba	25.5	0.56	0.30	40.9	330.1	1.11	0.21
*Potentilla erecta* (L.) Raeusch.	pot_erecta	51.5	0.34	0.29	39.3	80.1	0.62	0.60
*Primula elatior* Hill	primula	48.4	0.34	0.37	22.5	185.7	1.60	0.38
*Ranunculus acris* L.	ranunculus	130.3	0.17	0.25	26.1	24.3	0.59	2.37
*Rumex acetosa* L.	rumex	107.5	0.30	0.24	35.2	43.2	0.53	5.89
*Selinum carvifolium* (L.) L.	selinum	42.1	0.31	0.34	33.1	66.3	4.92	11.13
*Succisa pratensis* Moench	succisa	86.4	0.20	0.30	26.1	23.4	2.76	5.32
*Symphytum tuberosum* L.	symphytum	74.5	0.18	0.33	23.9	139.3	0.31	0.30
*Tanacetum vulgare* L.	tanacetum	30.7	0.21	0.46	42.0	111.8	2.83	11.44

Species names used follow the nomenclature used in the Pladias database (Chytrý *et al.*, 2021) and Key to the flora of the Czech Republic (Kaplan, 2019). Abbreviations: DMC, dry matter content; NA, missing data; RTD, root tissue density; SRL, specific root length.

### Fieldwork

In this paper, we use the terms ‘plant’, ‘individual’, ‘clonal fragment’ or ‘clone’ (such as in ‘whole-clone trait’) to mean a set of all the ramets physically interconnected by rhizomes. Fieldwork was carried out in several sessions, depending on the phenology of different species, from May to August 2021 and from March to September 2022. Prior to digging, the sites were inspected thoroughly, and a few plants were dug up solely for the purpose of examining the size and structure of their below-ground parts. Then, five individuals (with adjacent soil) were carefully dug up on each site (altogether, ten individuals per species). All plants were sampled in the same phenological stage: flowering only or flowering and fruiting. The excavated block of soil contained the whole rhizome and all the shoots originating from it. The top 25–30 cm of soil were explored for roots and rhizomes, accounting for >90 % of the below-ground organs of a given clone.

Excavated soil blocks were stored in plastic boxes and transported to the Institute of Botany in Třeboň for further handling within 48 h. Here, they were placed in basins of water and kept cool until washing. Saturating the soil block with water helped us considerably during the disentangling of the focal plant (clonal fragment) from neighbouring species and their root systems.

### Washing and above-ground measurements

During the washing, soil was carefully removed, as were the roots and shoots of neighbouring species. Nonetheless, some roots were lost during washing and handling of plant specimens. We only used the best-preserved individuals for fine root trait assessment, resulting in final counts of six to ten individuals per species. The individuals of study species were put into plastic cups with water, and within a few hours they were cut into the above-ground parts (i.e. leaves, stems and flowers) and the rhizome with roots. The above-ground parts were put into paper bags, dried at 60 °C for 24 h and weighed using analytical scales. The rhizomes with roots were stored in plastic cups with water in a refrigerator and handled within 48 h.

### Roots and rhizomes

**Fig. 1.**
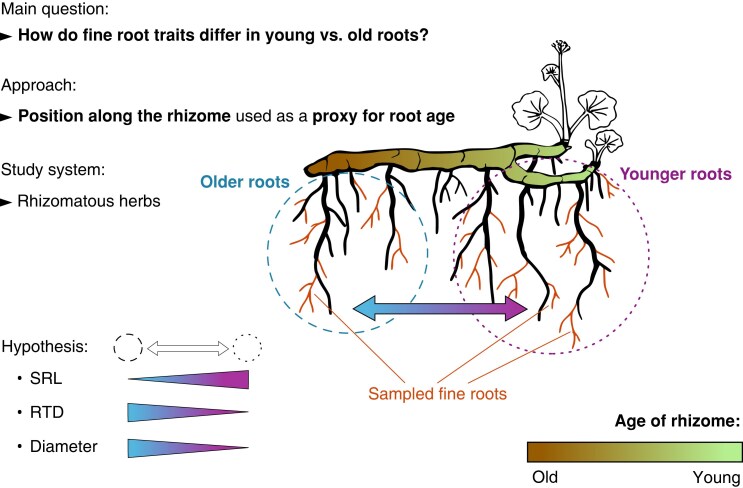
Conceptual scheme of the study. Abbreviations: RTD, root tissue density; SRL, specific root length.

Fine root samples were taken from young and old parts of the rhizome (for illustration, see Fig. 1), with the aim of maximizing the distance between root origin points. Therefore, the distance between the young and old part of a rhizome was relative and dependent on both rhizome length and the positions of vital roots. The criteria for choosing which roots to include in the fine root sample were common for all species. Only roots of the first three orders (i.e. three most distal orders) were used. In herbs, these orders can generally be categorized as absorptive fine roots (Holdaway *et al.*, 2011; Zhou *et al.*, 2022), following the functional classification (*sensu*  McCormack *et al.*, 2015), and their main function is water and nutrient acquisition (Zhou *et al.*, 2022). The roots from the absorptive fine root pool were chosen based on their appearance, namely colour and branching. Unbranched roots and roots damaged during processing were excluded from the sample. The assessment of suitability for trait measurement depended on species identity (e.g. in species with light-coloured roots, only light roots were used, as opposed to species with dark-coloured roots). Variation in appearance between roots from young and old parts was allowed. Only live roots were included in the sample, as corroborated by visual inspection of root anatomy using cross-sections of a few roots not included in the sample but similar in appearance.

The roots were placed in a Petri dish with a small amount of water and scanned immediately (flatbed scanner EPSON Perfection, 1200 dpi), then dried at 60 °C for 24 h and weighed using analytical scales. The rhizome length was measured as the total of all rhizome branches of a given clonal fragment. All remaining roots were cut off the rhizome, and the rhizome was weighed in the fresh state. Rhizomes were then dried at 60 °C for 48 h and weighed in the dry state using analytical scales for calculation of the rhizome dry matter content (rhizome DMC = dry weight/fresh weight).

### Root trait measurement

Images of the scanned roots were analysed using the software WinRhizo (Regent Instruments Inc., 2013). Data on root length, volume and dry weight were used to calculate specific root length [SRL = length/dry weight (in millimetres per milligram)] and root tissue density [RTD = dry weight/volume (in milligrams per millimetre cubed)]. Data on root diameter were obtained directly from the WinRhizo analysis.

### Data analysis and visualization

All data were analysed using R software (version 4.3.1; R Core Team, 2023). Prior to analyses, non-normally distributed variables were transformed using the natural logarithm or square root, with the aim of approaching a normal distribution as closely as possible. After transformation of non-normally distributed variables, a linear mixed-effect model (function *lmer* from the package *lme4*; Bates *et al.*, 2015) was used to determine the effect of rhizome age (i.e. young or old part), with sites and species as random factors. Separate linear mixed-effect models were carried out for each of the root traits measured, i.e. SRL, RTD and diameter. Model selection was carried out using the Akaike information criterion corrected for small sample size (function *AICc* from the package *MuMIn*; Bartoń, 2024). The function *rdacca.hp* from package *rdacca.hp* (Lai *et al.*, 2022) was used to calculate variation and hierarchical partitioning.

For assessment of the effect of whole-clone traits (rhizome length, rhizome DMC and above-ground biomass), we used ratios of root trait values from roots in old and young parts of the rhizome. These ratios were calculated at the level of an individual (clonal fragment) using the following formula: ratio = root trait value in old part of rhizome/root trait value in young part of rhizome. The effect of whole-clone traits on root trait ratios was assessed by similar procedure as mentioned above, using the function *lmer*, with the interaction between species and site as a random factor. After initial inspection, only the random intercept for species:site was used in these models, because the variance unique to species was estimated at zero or close to zero after accounting for site nested within species. Rhizome length and above-ground biomass (as fixed-effect predictors) were log-transformed in the respective models in order to account for the strongly right-skewed distribution.

Habitat conditions were estimated using EIVs (we used values adapted for the Czech flora by Chytrý *et al.*, 2018). We focused on EIVs for moisture and nutrients, calculating both the plain site mean (based on species presence) and the weighted site mean (based on species abundances). We calculated average root trait ratios for each site. Also, in order to determine whether the habitat conditions induced any shift in root trait values, we calculated average root trait values per site. Next, we analysed the association between either within-clone trait ratios or raw trait values and EIVs for moisture or nutrients, using both weighted and unweighted EIV means. Again, the function *lmer* was used, with species as a random factor and with added parameter weights (calculated as the inverse of the s.d. of the trait mean) to account for within-species variation.

We used the *rda* function from the package *vegan* (Oksanen *et al.*, 2019) to perform and visualize a principal component analysis (PCA). Data were standardized prior to the PCA using the *decostand* function, with the parameter method set to ‘standardize’, i.e. scaling the data into zero mean and unit variance. The ellipses were drawn using the *ordiellipse* function from the package *vegan*. The size and shape of the ellipses shown in PCA figures correspond to the s.d. of the values for a given species in the plane represented by the first two principal component (PC) axes, with the two most distant points on a given ellipse being oriented in the direction of largest variation within the mentioned plane.

We also used these packages for data manipulation and visualization: *corrplot* (Wei and Simko, 2024), *dplyr* (Wickham *et al.*, 2023*a*), *ggbeeswarm* (Clarke *et al.*, 2025), *ggplot2* (Wickham, 2016), *patchwork* (Pedersen, 2025), *stringr* (Wickham, 2023), *tidyr* (Wickham *et al.*, 2023*b*) and *tidyverse* (Wickham *et al.*, 2019). Incomplete observations were omitted from relevant analyses. One outlier was omitted from the dataset owing to the extreme size of both above-ground and rhizome, thus losing relevance in the context of our study.

## RESULTS

### Overall trait information

**Fig. 2.**
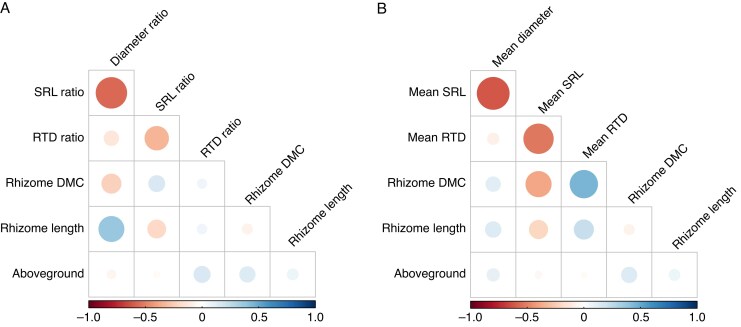
Correlation matrices of whole-clone traits in combination with root trait ratios (A) and mean root trait values (B). Circle size and colour saturation show the degree of correlation, and the hue represents the direction of correlation: red, negative; white, no correlation; blue, positive. Datasets are standardized whole-clone data for A and raw whole-clone data for B. Abbreviations: above-ground, above-ground biomass; DMC, dry matter content; RTD, root tissue density; SRL, specific root length.

We collected whole clonal fragments from a total of 174 individuals, representing 19 species across 25 localities. Mean fine root diameter ranged from 0.24 to 0.46 mm, mean SRL ranged from 25.5 to 130.3 m g^−1^, and mean RTD ranged from 0.17 to 0.56 g cm^−3^ (Table 1). SRL was negatively correlated with both RTD and diameter. We found no strong correlation between RTD and diameter. The negative association between SRL and diameter was the strongest among these correlations (Fig. 2B).

**Fig. 3.**
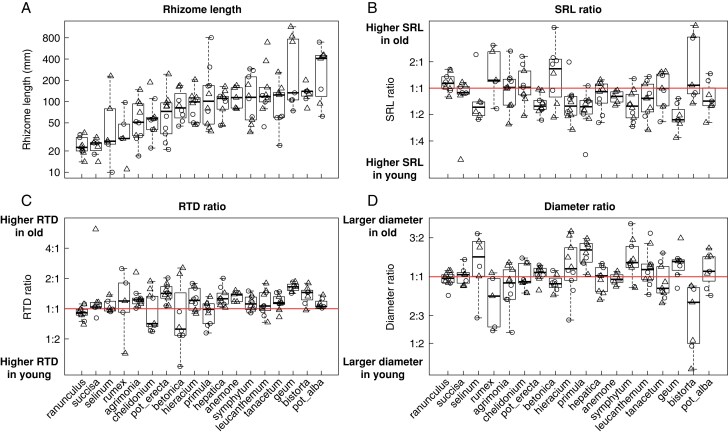
Rhizome length (A) and trait ratios (B–D) of the study species. Species are ordered by increasing rhizome length in all graphs. Red horizontal lines in B–D indicate a 1:1 ratio. For species abbreviations, see Table 1. The upper and lower ends of boxes indicate first and third quartiles, respectively; the horizontal line inside the boxes represents the median, and the ends of whiskers show minimum and maximum values apart from outliers (i.e. points further than 1.5 × interquartile range from box end). The *y*-axis is log-scaled in all four graphs. The shapes represent the two sites per species (triangles, site A; circles, site B). Abbreviations: RTD, root tissue density; SRL, specific root length.

Mean rhizome DMC ranged from ∼14 to ∼42 %, and mean rhizome length varied from ∼23 to ∼443 mm (Table 1; Fig. 3). Above-ground biomass showed substantial variation among species, changing by more than two orders of magnitude.

### Within-clone root trait variation

**Fig. 4.**
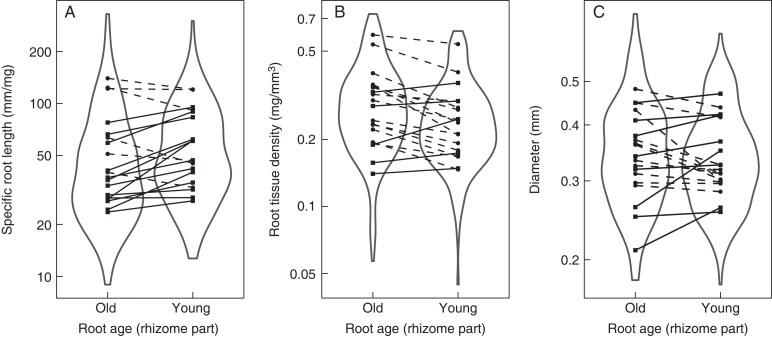
Fine root trait variation within rhizomes. Measured trait values of specific root length (A), root tissue density (B) and root diameter (C) in samples from young and old roots (rhizome parts). Violin-shaped outlines illustrate densities (numbers of observations) with respective values of traits. Black dots and lines represent means of trait values in old or young roots of a given species. The species with an increase in the mean from old to young are shown using squares and a continuous line; the species with a decrease in the mean from old to young roots are shown using filled circles and a dashed line. For detailed visualization the responses of root traits to root age (rhizome position) across all studied species, see Supplementary Data Figs S1– S3.

We found a small yet significant difference in SRL and RTD between roots in the young and old parts of a rhizome, with higher SRL in young part roots and higher RTD in old part roots (Fig. 4; Table 2). In some species (e.g. *Potentilla erecta*, *Geum rivale* or *Agrimonia eupatoria*), the trend was consistent across all individuals sampled at both localities (see Supplementary Data Figs S1 and S2). No significant difference between samples from the young and old parts of a rhizome was found for root diameter (Fig. 4).

**Table 2.** Effect of rhizome part (age) on root traits. Each row contains statistics from a mixed-effect model, with rhizome part (age) as a fixed effect, site and species as random effects, and the respective variable as a response variable.

Response variable	Estimate	s.e.	d.f.	*t*	*P*-value	Marginal	Conditional
log(SRL)	**0.17791**	**0.06963**	**18.64**	**2.555**	0.0195*	0.016	0.680
sqrt(RTD)	**−0.03903**	**0.01140**	**18.67**	**−3.424**	0.0029**	0.028	0.736
log(diameter)	−0.01649	0.03117	18.10	−0.529	0.603	0.001	0.649

Abbreviations: conditional, variation explained by fixed + random effect; marginal, variation explained by fixed effect; RTD, root tissue density; SRL, specific root length. Significant effect of root age on a trait is indicated by bold text and asterisks next to *P*-values (*, alpha = 0.05; **, alpha = 0.01).

For further exploration of trait differences between fine roots from old and young parts of a rhizome, we used trait ratios. Species identity, followed by collection site, explained most of the variation in all the root traits observed (see Tables 2 and 3). The effects of species and site overlapped markedly in the cases of all three fine root traits.

**Table 3.** Variation partitioning of root traits.

Response variable	*R* ^2^	Part^a^ (%)	Site^a^ (%)	Species^a^ (%)
log(SRL)	0.561	2.9	39.6	57.5
sqrt(RTD)	0.692	4.3	44.6	51.1
log(diameter)	0.541	0	35.5	64.5

The table shows data obtained using *rdacca.hp*, a combination of variation partitioning and hierarchical partitioning based on a redundancy analysis (RDA). Abbreviations: R^2^, total explained variation by the predictor variables (obtained from canonical analysis RDA); RTD, root tissue density; SRL, specific root length. ^a^Numbers shown in these columns are percentages of the total *R*^2^ value explained by the respective predictor.

### Effect of whole-clone traits on root trait ratios and mean values

**Fig. 5.**
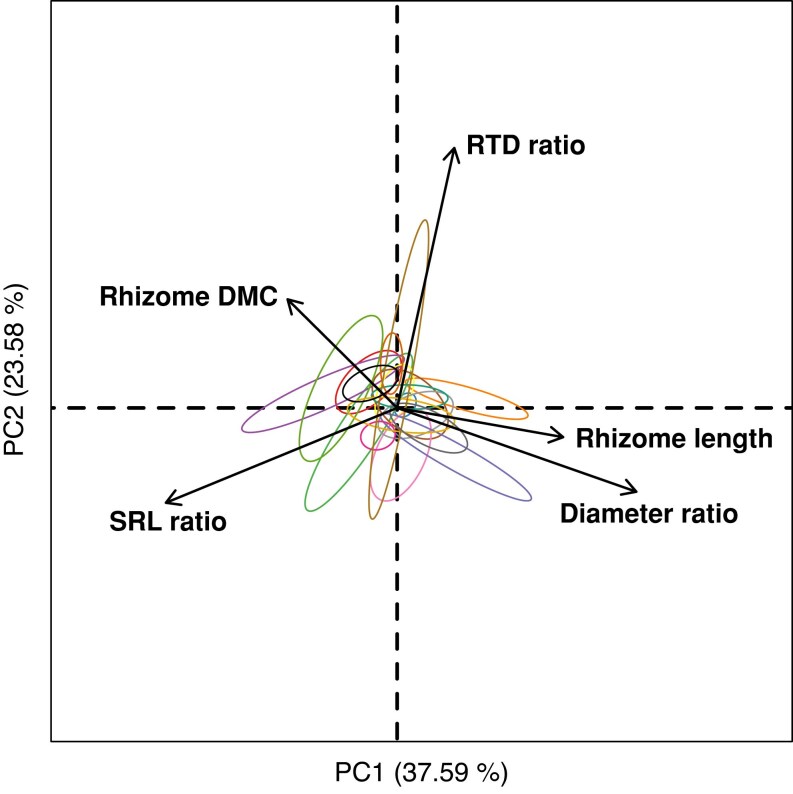
Principal component analysis of rhizome traits and root trait ratios (calculated as old roots:young roots). Values in parentheses in axis labels show explained variation by axis (out of total unconstrained variation). Ellipses represent different species and show degree and directions of trait variation along the two principal component (PC) axes.

Rhizome length was negatively correlated with the SRL ratio and positively correlated with the diameter ratio (Figs 2A and 5; Table 4). This means that relative root trait differences between old and young roots increased with increasing rhizome length. Although we observed no significant root trait variation along rhizomes of short-rhizome plants, plants with long rhizomes tended to feature roots of lower diameter and higher SRL in the young part in comparison to roots in the old part. The RTD ratio was not affected by rhizome length. Rhizome length exhibited a marginally negative association with mean SRL (*P* = 0.064, *R*^2^ = 0.019) and a positive association with mean root diameter (*P* = 0.007, *R*^2^ = 0.047).

**Table 4.** Effect of whole-clone traits on fine-root trait ratios.

Fixed effect	Response variable	Estimate	s.e.	d.f.	*t*	*P*-value	Marginal	Conditional
log(rhizome length)	**log(SRL ratio)**	**−0.190**	**0.055**	**108.1**	**−3.433**	0.0008***	0.093	0.372
	log(RTD ratio)	0.046	0.037	111.9	1.246	0.215	0.013	0.341
	**log(diameter ratio)**	**0.074**	**0.024**	**124.3**	**3.078**	0.003**	0.074	0.465
rhizome DMC	log(SRL ratio)	1.313	0.733	66.1	1.792	0.078	0.034	0.357
	log(RTD ratio)	0.197	0.474	66.3	0.416	0.678	0.002	0.347
	**log(diameter ratio)**	**−0.774**	**0.315**	**72.8**	**−2.453**	0.017*	0.064	0.444
log(above-ground biomass)	log(SRL ratio)	−0.012	0.042	65.8	−0.295	0.769	0.001	0.349
	log(RTD ratio)	0.018	0.027	68.9	0.694	0.490	0.005	0.357
	log(diameter ratio)	0.000	0.018	76.6	0.015	0.988	0.000	0.425

Each row contains statistics from a mixed-effect model, with a given variable as a fixed effect (first column), site and species as random effects, and a respective variable (second column) as a response variable. Abbreviations: conditional, variation explained by fixed + random effect; DMC, dry matter content; marginal, variation explained by fixed effect; RTD, root tissue density; SRL, specific root length. Significant results are indicated by bold text and asterisks next to *P*-values (*, alpha = 0.05; **, alpha = 0.01; ***, alpha = 0.001).

Correlations between fine root trait ratios and either rhizome DMC or total above-ground biomass were rather weak (see Fig. 2A). Rhizome DMC had no significant effect on the SRL and RTD ratios but had a marginal effect on diameter ratio (Table 4). Total above-ground biomass did not affect the ratios of any of the root traits measured (Table 4).

Rhizome DMC was correlated with mean SRL and RTD values (see Fig. 2B). When tested, rhizome DMC showed a significant negative effect on mean SRL (*P* = 0.050, *R*^2^ = 0.041) and a positive effect on mean RTD (*P* = 0.002, *R*^2^ = 0.085). Above-ground biomass did not affect mean trait values (within-clone averages) of root traits.

### Effect of habitat conditions on root trait values and ratios

We observed no significant response of trait ratios to site moisture or nutrient status proxied by EIVs (Supplementary Data Tables S2 and S3). Mean values of root traits or whole-clone traits were not affected by the EIVs. In three cases, the trait ratios seemed to be correlated with EIVs for either moisture or nutrients, but closer inspection of the models showed that in all three cases the models were biased by single outlier data points with high leverage. Without these points, the relationships were no longer significant.

## DISCUSSION

In this study, we investigated how three fine root morphological traits change during root lifespan (using root position along the rhizome as a proxy of root age). Although most of the variation in fine root morphology was driven by species identity and the site of plant collection, we also found a small effect of root ontogeny on all the traits. We observed lower RTD and higher SRL in young compared with old fine roots. Variation of both root diameter and SRL depended on rhizome length; the longer the rhizome, the larger the differences between young and old fine roots. Likewise, root diameter variation was more pronounced in plants with higher rhizome DMC. Site moisture and nutrient conditions (estimated by EIVs) had no effect on mean root trait values per individual or on the differences in root trait values between young and old fine roots. On balance, our findings show that the variation in fine root traits within individuals was much smaller than the variation among species. Therefore, in herbs with short to mid-sized rhizomes, sampling fine roots in different positions along rhizomes does not introduce any substantial bias.

### Patterns of root trait shifts

We believe that the observed patterns of SRL (smaller in older roots), RTD and root diameter (both larger in older roots) changes in our study can be attributed to trait shifts during root lifespan. An increase of RTD during plant ontogeny was previously observed by Pastor-Pastor *et al.* (2021). Shifts of root diameter and SRL became evident (or more pronounced) once rhizome length had been considered, probably because in longer rhizomes the differences in root age were larger. Analogously, within-clone differences in root diameter could be observed in plants with higher rhizome DMC. These effects are likely to be attributable to longer and drier rhizomes being older (Lubbe *et al.*, 2023) and thus probably featuring roots of more diverse age. Other studies found similar development in SRL and root DMC (Tobner *et al.*, 2013; Havrilla *et al.*, 2021) with increasing age of whole individuals. In our study, roots in the young part were also often more branched than those in the old part (Hrouda *et al.*, 2026; A Hrouda pers. obs.), which also aligns with the findings of Tobner *et al.* (2013), who showed that SRL and branching intensity were positively associated. The increase in diameter might have been caused by root thickening, a natural phenomenon during root ontogeny of dicots (Hishi, 2007; Zhou *et al.*, 2022), which increases the transport capacity of roots. It is also possible that in the old root samples, more of the finest (first or second order) roots had been lost during growth, resulting in increase of average diameter. Such an increase in diameter would probably become evident only over longer time periods, i.e. in plants with longer-lived rhizomes.

On a larger scale, higher SRL and lower RTD are often associated with more efficient nutrient uptake (Eissenstat, 1992; Bouma *et al.*, 2001; Hou *et al.*, 2024). The ontogenetic shift in root traits described here could indicate that the main function of young roots is acquisitive and that with ageing it changes towards transport. Overall, however, trait changes within individuals were small in magnitude in comparison to interspecies differences. Based on that, we cannot deduce that the differences in traits were accompanied by a modification of function. We infer that such fine root trait shifts need not be considered in large-scale analyses, nor is it important to reflect them in standardized root trait measurement protocols. We conclude that fine root morphological trait changes caused by ontogeny might play a role only in small scale processes within the rhizosphere.

It is important to bear in mind that although changes in morphology during root lifespan were small, undetected changes might have been happening on a physiological level. Nutrient uptake abilities have been reported to change the most in the early stage of the root lifespan and later continue to decrease only slightly (Wells and Eissenstat, 2002; Hishi, 2007). Therefore, the differences in nutrient uptake between very young roots and old roots might be pronounced. In a study on root foraging in seedlings, van Vuuren *et al.* (1996) observed both morphological (later) and physiological (earlier) responses to soil nutrient heterogeneity. Fransen *et al.* (1999) used parts of a mature clone and found a physiological but no morphological reaction to a nutrient-rich patch. Owing to the scarcity and varying scope of similar studies, it remains unclear how long fine roots retain their plasticity in various physiological traits and whether any universal trends across species exist. In future research, combined observation of both physiology and morphology might broaden our understanding of root dynamics and functioning.

### Root age along the rhizome

Owing to methodological difficulties, there are few existing studies about the effect of position in a root system (Klimešová and Herben, 2023) or root age on fine root traits (Majdi *et al.*, 2001; Hishi, 2007). Our study is the first to show that some root traits of rhizomatous herbs differ between young and old parts of a rhizome and that the differences are affected, in part, by the rhizome length. We were working with the assumption that roots in the young part of a rhizome were younger than roots in the old part, although we did not measure the age of the roots directly. Fine roots of several temperate herb species can live for more than one season (Ryser and Kamminga, 2009; Courchesne *et al.*, 2020; Lubbe *et al.*, 2021), allowing for the possibility of substantial variation in root age within a root system. The shifts of SRL, RTD and diameter, together with decreased branching of roots in the old part of rhizome, observed in our study suggest that root age did indeed vary, because in roots of the same or similar age such changes would be improbable. Although our design does not allow us to disentangle the effects of root age and rhizome age (i.e. part of rhizome) completely, root age seems to be an important driver of the observed morphological variation.

Furthermore, the fact that the pattern of fine root traits shifts between roots of varying age (and position) was consistent deserves further attention. Even if the morphological changes were small, they highlight the co-occurrence of roots in different stages of their lifespan, and therefore different potential to respond plastically to current conditions. Sun *et al.* (2016) observed that at the time when lateral roots of a rooting unit started to die off, new lateral roots were no longer produced on the same rooting unit. Also, in a simulation study by Dunbabin *et al.* (2004), the efficiency of nitrate uptake was linked to the capacity to proliferate roots, a characteristic of early to mid-life stages of a root. On balance, the younger roots within a root system might therefore have a big advantage in terms of adaptability in comparison to the older roots.

### Study conditions and challenges

Our field study approach enabled us to examine trait variation in root ontogeny by working with mature plant individuals. It allowed us to compare roots originating from young and old parts of an established rhizome, and arguably, it included plant individuals that were older and had more developed rhizome systems than would have been possible within a greenhouse experiment. Indeed, the effect of ontogeny is often hard to observe in greenhouse experiments owing to their short duration (Alvarez-Flores *et al.*, 2014; Garbowski *et al.*, 2021; Torres *et al.*, 2022). At the same time, conducting the experiment in natural conditions introduced some potential confounding factors, such as size differences, environmental gradients or soil heterogeneity. That being said, we found no link between the above-ground biomass and fine root trait differences between young and old roots or between site moisture or nutrients and either mean values of fine root traits or the ratio of trait values between young and old roots. Although environmental gradients can affect the traits we observed (Fort and Freschet, 2020), in our study the difference in EIVs between two sites of the same species was rather small, suggesting that there was no substantial variation to respond to. Also, soil characteristics, such as soil bulk density (Freschet *et al.*, 2017) and nutrient heterogeneity (Březina *et al.*, 2019), are often found to affect fine root traits (Hodge, 2004). Based on our observation (without measurement), soils at the two sites of the same species were generally comparable in density. Any foraging response based on heterogeneity is unlikely, because our collection sites were often meadows, and the shallow below-ground layers there were densely filled with roots (Hutchings *et al.*, 2003; Březina *et al.*, 2019).

### Conclusion

We found that fine root morphological traits of rhizomatous herbs undergo changes throughout root lifespan. This can lead to trait variation within a root system, especially in plants with longer rhizomes. We also found that the magnitude of trait variation related to age (or position) is much smaller than interspecies differences. Thus, although ontogenetic trait variation might play a role in small-scale soil resource depletion or competition, its effect on species average trait values is small. Fine roots of herbaceous plants deserve further research that would provide insights into trait development of fine roots and the effects of fine root position in root systems.

## Supplementary Material

mcag103_Supplementary_Data

## Data Availability

The data that support the findings of this study are openly available in Mendeley Data depository at http://doi.org/10.17632/d37jrnfcvb.1.

## SUPPLEMENTARY DATA

Supplementary information contains two documents: Supplementary Tables and Supplementary Figures. Supplementary Tables consist of a table of Ellenberg indicator values (EIVs) of collection sites (Supplementary Data Table S1) and tables with test statistics from analyses of effect of EIVs on root traits (Supplementary Data Tables S2 and S3). Supplementary Figures consist of detailed graphs showing root trait variation with root age in different species for specific root length (Supplementary Data Figure S1), root tissue density (Supplementary Data Figure S2) and root diameter (Supplementary Data Figure S3). Table S1: Ellenberg indicator values (EIVs) of collection sites. Values in columns marked as ‘plain’ are calculated based on species presence only. Values in columns marked as ‘weighted’ are calculated using species abundances, with higher weight given to more abundant species. Table S2: effect of habitat conditions on root trait ratios. Each row contains statistics from a mixed-effect model, with a given variable as a fixed effect (first column), species as a random effect, and a respective variable (second column) as a response variable. Asterisk in parentheses (*) indicates significant effect (alpha = 0.05) driven by a single outlier; the omission of the outlier resulted in the effect becoming non-significant. Abbreviations: conditional, variation explained by fixed + random effect; d.f., degrees of freedom; marginal, variation explained by fixed effect; *P*, *P*-value; s.e., standard error; *t*, *t*-value. Table S3: effect of habitat conditions on mean root trait values. Each row contains statistics from a mixed-effect model, with a given variable as a fixed effect (first column), species as a random effect, and a respective variable (second column) as a response variable. Mean root trait values were calculated as within-clone averages. Abbreviations: conditional, variation explained by fixed + random effect; d.f., degrees of freedom; marginal, variation explained by fixed effect; *P*, *P*-value; s.e., standard error; *t*, *t*-value. Figure S1: variation of specific root length between old and young roots among 19 species. Coloured symbols represent means for the respective combination of species and root age; blue circles are means for old roots, and violet triangles are means for young roots. The error bars show ±1 s.d. calculated from log-transformed values and back-transformed. See Table 1 for list of species abbreviations. Figure S2: variation of root tissue density between old and young roots among 19 species. Coloured symbols represent means for the respective combination of species and root age; blue circles are means for old roots, and violet triangles are means for young roots. The error bars show ±1 s.d. calculated from log-transformed values and back-transformed. See Table 1 for list of species abbreviations. Figure S3: variation of root diameter between old and young roots among 19 species. Coloured symbols represent means for the respective combination of species and root age; blue circles are means for old roots, and violet triangles are means for young roots. The error bars show ±1 s.d. calculated from log-transformed values and back-transformed. See Table 1 for list of species abbreviations.
