# Supplementary material for: The position along rhizome as a surrogate for root age: effect on fine root traits
Source: Ann Bot. 2026 Apr 18;138(2):357–67. doi: 10.1093/aob/mcag103 (PMC13454676; doi:10.1093/aob/mcag103)

Figure S1: Variation of specific root length between old and young roots among 19 species. Coloured symbols represent means for the respective combination of species and root age; blue circles are means for old roots and violet triangles are means for young roots. The error bars show ±1 SD calculated from log-transformed values and back-transformed. See Table 1 for list of species abbreviations.

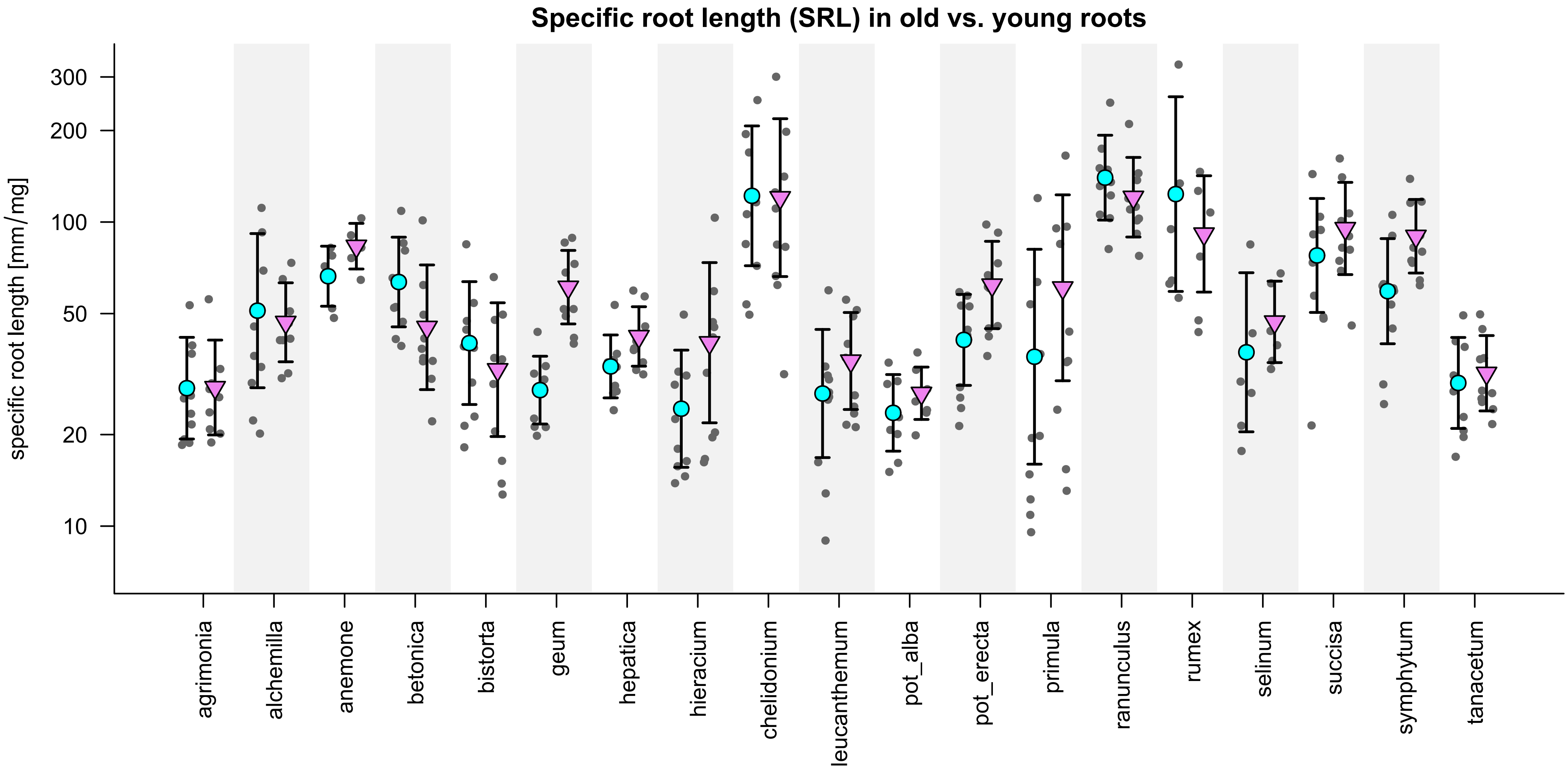

Figure S2: Variation of root tissue density between old and young roots among 19 species. Coloured symbols represent means for the respective combination of species and root age; blue circles are means for old roots and violet triangles are means for young roots. The error bars show ±1 SD calculated from log-transformed values and back-transformed. See Table 1 for list of species abbreviations.

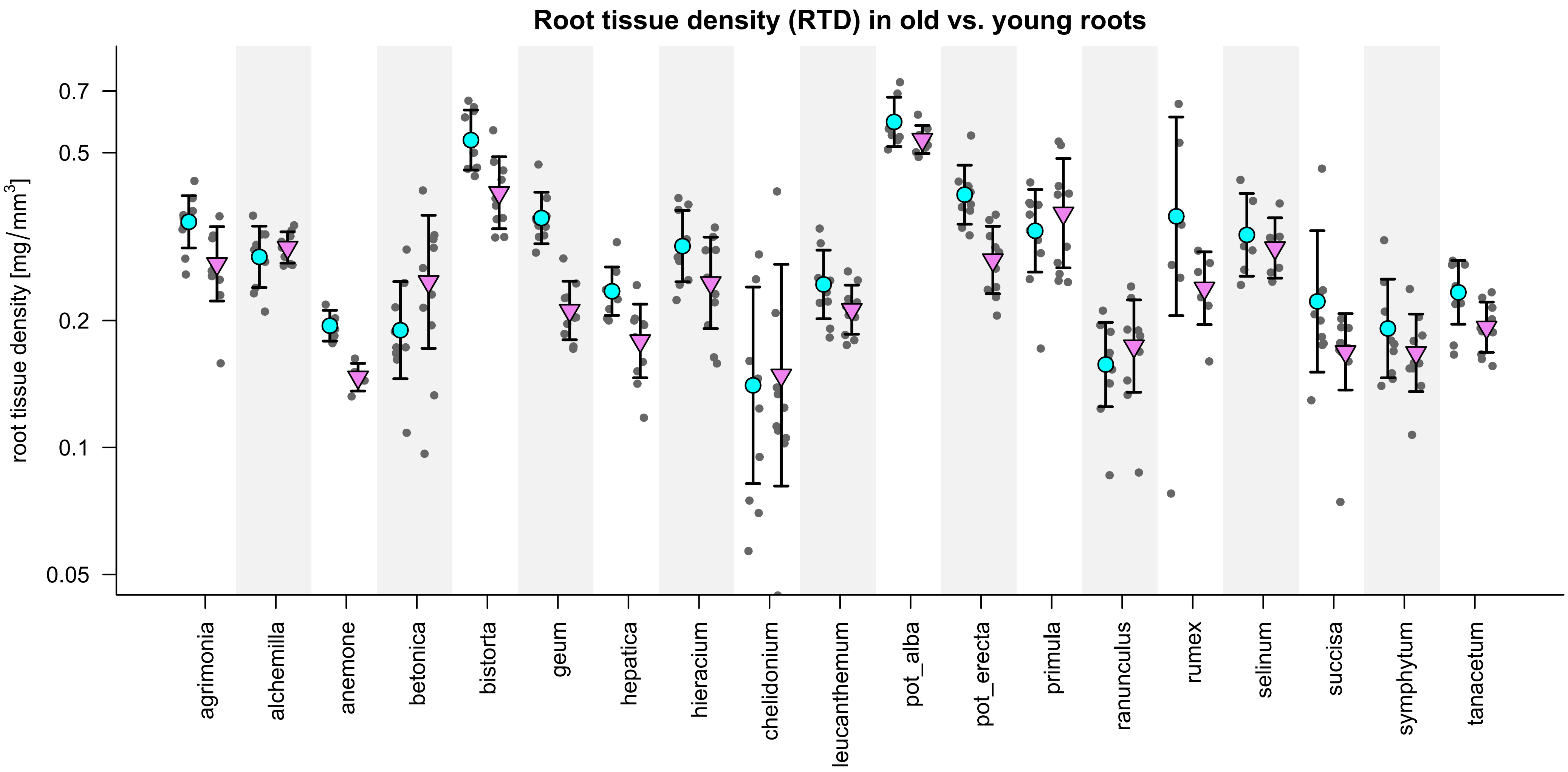

Figure S3: Variation of root diameter between old and young roots among 19 species. Coloured symbols represent means for the respective combination of species and root age; blue circles are means for old roots and violet triangles are means for young roots. The error bars show ±1 SD calculated from log-transformed values and back-transformed. See Table 1 for list of species abbreviations.

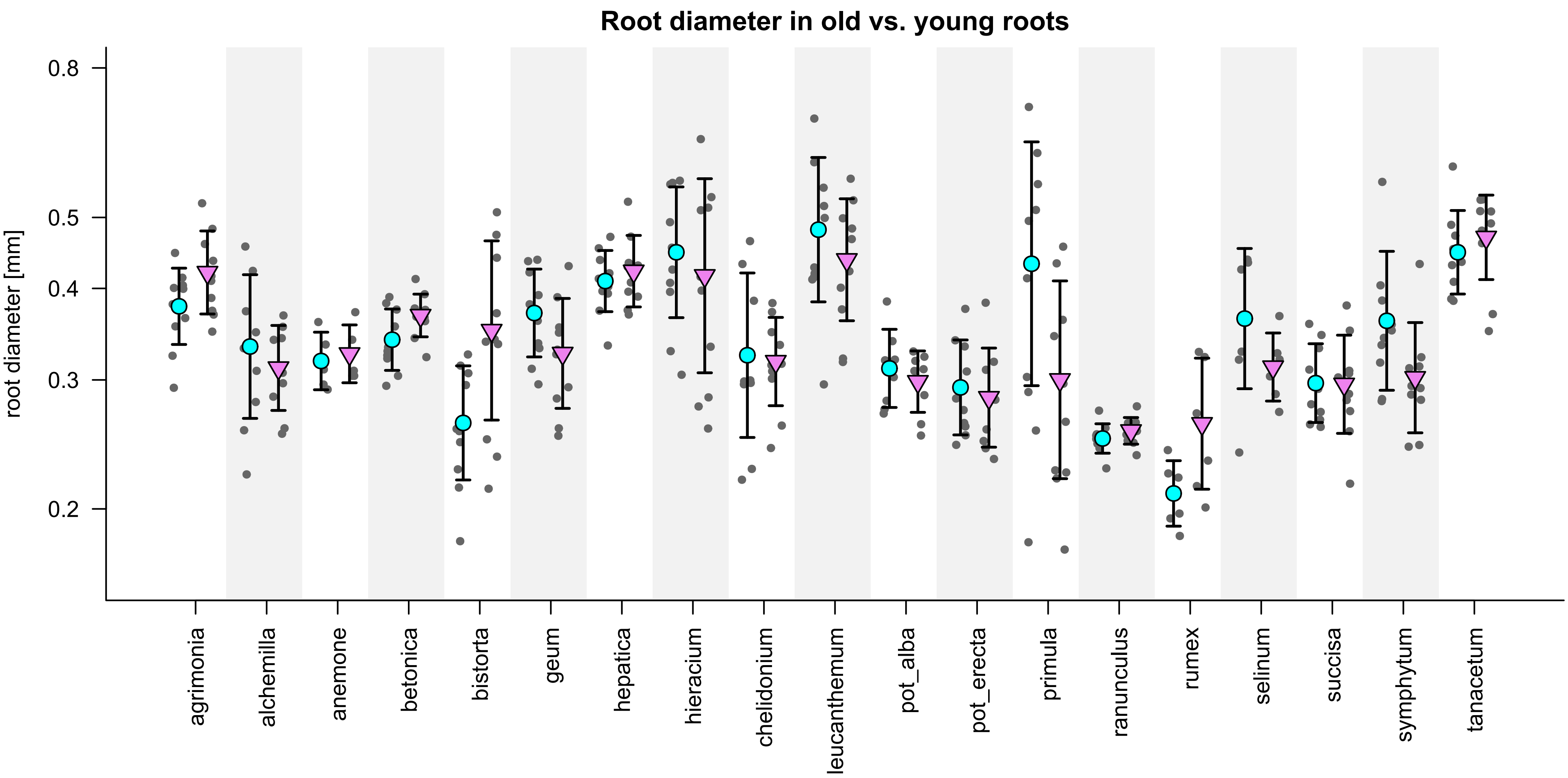

Supplement: Supplementary file 1 [file mcag103_supplementary_data.zip › Supplementary_Figures_PDF.pdf]
