# Supplementary material for: The position along rhizome as a surrogate for root age: effect on fine root traits
Source: Ann Bot. 2026 Apr 18;138(2):357–67. doi: 10.1093/aob/mcag103 (PMC13454676; doi:10.1093/aob/mcag103)
Supplement: Supplementary file 1 [file mcag103_supplementary_data.zip › Supplementary_Tables_PDF.pdf]

Table S1: Ellenberg indicator values (EIVs) of collection sites. Values in columns marked as ‘plain’ are calculated based on species presence only. Values in columns marked as ‘weighted’ are calculated using species abundances, with higher weight given to more abundant species.

species	**moisture** (plain)	**moisture** (weighted)	**nutrients** (plain)	**nutrients** (weighted)	site description
Agrimonia eupatoria	5.16	5.25	5.84	5.74	wet meadow near Posměch pond
	5.65	5.96	5.45	6.01	near Třeboň, unmowed grassland in Branná
Alchemilla glaucescens	5.07	5.14	5.66	5.53	Radkov u Telče, grassland edge
	4.84	4.85	4.81	4.83	Jiříkovo valley, grassland slope, former field
Anemone nemorosa	6.21	5.29	5.79	5.29	Stříbřec, Nová řeka river, near Stříbřecký bridge
Betonica officinalis	5.57	5.09	4.96	4.76	České Budějovice, Vrbenské ponds
	6.43	5.89	4.21	4.15	Lužnice, Černiční pond
Bistorta officinalis	5.91	6.02	5.12	5.42	Železné hory
	5.38	5.43	5.05	5.09	Železné hory, Soběnov
Geum rivale	5.76	6.59	6.12	5.98	Železné hory
	7.45	7.76	5.27	5.05	Železné hory
Hepatica nobilis	5.38	5.21	6.25	6.74	Libníč, forested abandoned quarry
	4.76	4.62	4.92	4.22	Vidov, forest slope
Hieracium murorum	5.00	4.97	4.41	3.54	Byňov, forest clearing N of road
	5.50	4.78	4.83	4.20	Staňkov, near west bank of Staňkovský pond
Chelidonium majus	5.38	6.63	6.08	4.63	Třeboň, Hrádeček
	5.11	5.06	7.33	7.96	Třeboň, Klec
Leucanthemum ircutianum	5.03	4.96	5.12	5.23	Jiříkovo valley, grassland, former field
	4.61	4.81	4.94	5.73	Libníč, grassland above Dobrá Voda stream

Potentilla alba	4.10	4.02	3.93	3.91	Bílé Karpaty mountains, drier part of grassland
	4.85	4.98	4.54	4.51	Bílé Karpaty mountains, wetter part of grassland
Potentilla erecta	5.45	5.34	4.18	3.52	České Budějovice, Vrbenské ponds
	6.45	6.01	4.38	3.91	Pístina
Primula elatior	6.85	7.35	5.65	5.18	Velechvín forest area, along a stream
	5.93	5.85	6.25	6.67	Koloměřice, river bank of Bolíkovský stream
Ranunculus acris	6.58	6.84	5.32	5.16	Třeboň, Mokré louky
	5.22	5.27	5.57	5.97	Vlkovice
Rumex acetosa	6.58	6.84	5.32	5.16	Třeboň, Mokré louky
	5.22	5.27	5.57	5.97	Vlkovice
Selinum carvifolia	6.23	4.74	5.38	4.35	Lužnice, shore of Černičný pond
	6.67	6.23	5.33	5.76	Lužnice, shore of Černičný pond, wetter part
Succisa pratensis	6.00	4.97	4.79	4.44	Lužnice, shore of Černičný pond
	5.59	5.18	4.43	4.70	Pístina, wet meadow slope near Záhlinský pond
Symphytum tuberosum	5.26	5.27	5.97	6.37	Libníč, slope in Dobrá Voda stream valley
	4.76	4.62	4.92	4.22	Vidov, forest slope
Tanacetum vulgare	5.09	5.17	5.55	5.70	Lužnice
	5.46	5.67	6.07	6.20	Třeboň

Table S2: effect of habitat conditions on root trait ratios. Each row contains statistics from a mixed-effect model, with a given variable as a fixed effect (first column), species as a random effect, and a respective variable (second column) as a response variable. Asterisk in parentheses (*) indicates significant effect (alpha = 0.05) driven by a single outlier; the omission of the outlier resulted in the effect becoming non-significant. Abbreviations: conditional, variation explained by fixed + random effect; d.f., degrees of freedom; marginal, variation explained by fixed effect; *P*, *P-value*; s.e., standard error; *t*, *t-value*.

**fixed effect**	**response variable**	**estimate**	**s.e.**	**d.f.**	***t***	***P***	**marginal**	**conditional**
**moisture EIV**	log (SRL ratio)	-0.248	0.111	25.6	-2.223	0.035 (*)	0.128	0.821
	log (RTD ratio)	0.148	0.096	30.9	1.535	0.135	0.133	0.615
	log (diameter ratio)	0.091	0.046	28.7	1.987	0.057	0.104	0.931
**moisture EIV (weighted)**	log (SRL ratio)	-0.145	0.094	26.6	-1.533	0.137	0.068	0.773
	log (RTD ratio)	0.066	0.085	30.8	0.78	0.441	0.04	0.569
	log (diameter ratio)	0.061	0.036	28	1.708	0.099	0.067	0.928
**nutrients EIV**	log (SRL ratio)	0.034	0.143	30.7	0.239	0.813	0.003	0.726
	log (RTD ratio)	-0.187	0.08	22.3	-2.347	0.028 (*)	0.254	0.528
	log (diameter ratio)	0.008	0.059	30	0.133	0.895	0.001	0.919
**nutrients EIV (weighted)**	log (SRL ratio)	-0.064	0.083	27.1	-0.764	0.451	0.021	0.73
	log (RTD ratio)	-0.051	0.062	30.6	-0.83	0.413	0.041	0.487
	log (diameter ratio)	0.007	0.031	23.5	0.233	0.817	0.001	0.919

Table S3: effect of habitat conditions on mean root trait values. Each row contains statistics from a mixed-effect model, with a given variable as a fixed effect (first column), species as a random effect, and a respective variable (second column) as a response variable. Mean root trait values were calculated as within-clone averages. Abbreviations: conditional, variation explained by fixed + random effect; d.f., degrees of freedom; marginal, variation explained by fixed effect; *P*, *P-value*; s.e., standard error; *t*, *t-value*.

**fixed effect**	**response variable**	**estimate**	**s.e.**	**d.f.**	***t***	***P***	**marginal**	**conditional**
**moisture EIV**	log (SRL)	-0.059	0.107	27.905	-0.552	0.586	0.001	0.081
	log (RTD)	0.101	0.059	25.582	1.713	0.099	0.046	0.984
	log (diameter)	-0.061	0.051	34.998	-1.194	0.241	0.067	0.96
**moisture EIV (weighted)**	log (SRL)	-0.068	0.081	25.278	-0.844	0.407	0.001	0.089
	log (RTD)	0.005	0.046	24.933	0.098	0.923	0	0.979
	log (diameter)	-0.037	0.031	27.941	-1.218	0.234	0.051	0.956
**nutrients EIV**	log (SRL)	0.169	0.117	33.871	1.438	0.16	0.005	0.075
	log (RTD)	-0.081	0.078	34.789	-1.038	0.306	0.034	0.975
	log (diameter)	-0.032	0.048	34.193	-0.665	0.51	0.026	0.935
**nutrients EIV (weighted)**	log (SRL)	0.033	0.062	24.009	0.542	0.593	0	0.073
	log (RTD)	0.071	0.053	26.481	1.32	0.198	0.03	0.981
	log (diameter)	0.045	0.038	32.726	1.179	0.247	0.047	0.964
